# A human factor–centric validation of a security management system in a linked critical infrastructure environment

**DOI:** 10.1007/s42454-025-00070-2

**Published:** 2025-09-11

**Authors:** Florian Piekert, Tim H. Stelkens-Kobsch, Meilin Schaper, Hilke Boumann, Nils Carstengerdes

**Affiliations:** Institute of Flight Guidance, German Aerospace Center DLR, 38108 Brunswick, Germany

**Keywords:** Validation, Human factors, Security management, Linked critical infrastructures

## Abstract

This work reports the human factors–related validation results of a security system for the protection of linked critical infrastructures against combined cyber-physical attacks conducted in the European Horizon 2020 project PRAETORIAN (Protection of Critical Infrastructures from advanced combined cyber and physical threats). In order to prevent or mitigate interruption of services to the public, the protection of critical infrastructures is of high importance. The PRAETORIAN toolset is specifically designed to support security managers of critical infrastructures in their decision-making processes. It enables them to anticipate, manage, and withstand potential cyber, physical, or combined security threats that could target their own infrastructures or interconnected critical infrastructures. These threats could have a substantial impact and potentially compromise the safety and security of the population residing in their vicinities. The toolset consists of four primary systems: the physical, the cyber and the hybrid situation awareness and the coordinated response system. Each system is composed of different modules. Central to the toolset is the interconnecting interoperability platform. This interconnection facilitates seamless information exchange across all systems’ modules, ensures efficient data storage, prevents data duplication and inconsistencies, and replicates any changes made. The focus of the validation was put on the operators’ feedback assessment. In four exercises, attack scenarios were presented to groups of operators along with demonstrations of the PRAETORIAN tools. Feedback was collected using questionnaires, debriefing, and open questions. Key validation results show that the system offers benefits for cross-infrastructure security management, but improvements to systems and human–machine interfaces, procedures, and responsibilities are required.

## Introduction

In recent years, the society was repeatedly struck by diverse security incidents. To mitigate their impact, there is a need to anticipate, detect, and manage them. While such events happen and after suffering from them, it is of utmost importance that vital services for society are kept operational and secured. The bomb attacks in Brussels directed at the airport and a metro station in 2016 (BBC News 2016) are just examples for interconnected, coordinated, and increasingly more complex acts of terrorism. Adding up to this, there is a threat that is largely unrecognised by the general public despite its risk potential: The risk posed by a cyber and/or physical attack on a critical infrastructure (CI) that extends beyond the owners and operators of the targeted assets. It also encompasses their suppliers, customers, businesses, and individuals in close proximity of the infrastructure. Moreover, CIs that are linked due to nowadays increasing connectivity can be negatively impacted by such an attack. The consequences of an attack on a single CI can be extensive and have far-reaching effects on multiple sectors of the economy. In this paper, the term ‘linked CIs’ will be used to describe all instances in which events at one CI could affect another CI in any way.

For instance, there was an attack in 2016 that resulted in destruction of computers across six Saudi Arabian organisations, including energy, manufacturing, and aviation sectors (Pagliery 2016). Additionally, the WannaCry Ransomware attack, which occurred in 2017, impacted over 100,000 organisations in 150 countries (Norton Rose Fulbright 2017). Ukraine experienced vast power outages in both 2015 and 2017 (Zetter 2016) and not to forget the attack on the New York Dam in 2013 (Connor et al. 2015). Malware attacks like NotPetya in 2017 cause damages in the order of billions (Greenberg 2018) while the largest Distributed Denial of Service (DDoS) attack in the world targeted GitHub in 2018 (Ranger 2018).

Combined cyber and physical attacks on linked CIs will likely continue to rise. Several reasons contribute to this trend, as for example the five mentioned in Gooch (2020): (i) they already happened, (ii) there is a proliferation of industrial control system malware, (iii) there is an increased reliance of industry and CIs on information and communications technology (ICT) systems, (iv) industrial control system networks are notoriously difficult to secure, (v) cyber criminals have a proven business model. Adding to this, the availability of generative Artificial Intelligence (AI) opens up a completely new area of application for adversaries. Furthermore, attackers are eager to take advantage of situations that leave a country weak and defenceless, e.g., during an already on-going attack, natural disasters, or pandemic events. Some recent examples are the series of attacks during the pandemic situation caused by COVID-19, e.g., the Ripple20 vulnerabilities impacting the communications of millions of Internet of Things (IoT) medical devices (Davis 2020), cyber-attacks to vaccine test centres (Winder 2020), or ransom demands to hospitals (Gallagher and Bloomberg 2020).

In order to support successful handling of such critical situations, several projects have been funded by the European Commission during the last decades. First these were focused on securing specific domains and CIs. For example, the project GAMMA (Asgari et al. 2017) addressed security management in the domain of air traffic management (ATM), and the project SATIE (Stelkens-Kobsch et al. 2021) was focused on airports. Later on, a more holistic approach that considers CIs as linked systems was taken. Among the latter kind of projects is PRAETORIAN (Guyomard and Rigal 2022; Papadopoulos et al. 2023), which was designed to enhance the security and resilience of European CIs by aiding the coordinated protection of linked CIs against combined physical and cyber threats.

**Fig. 1:**
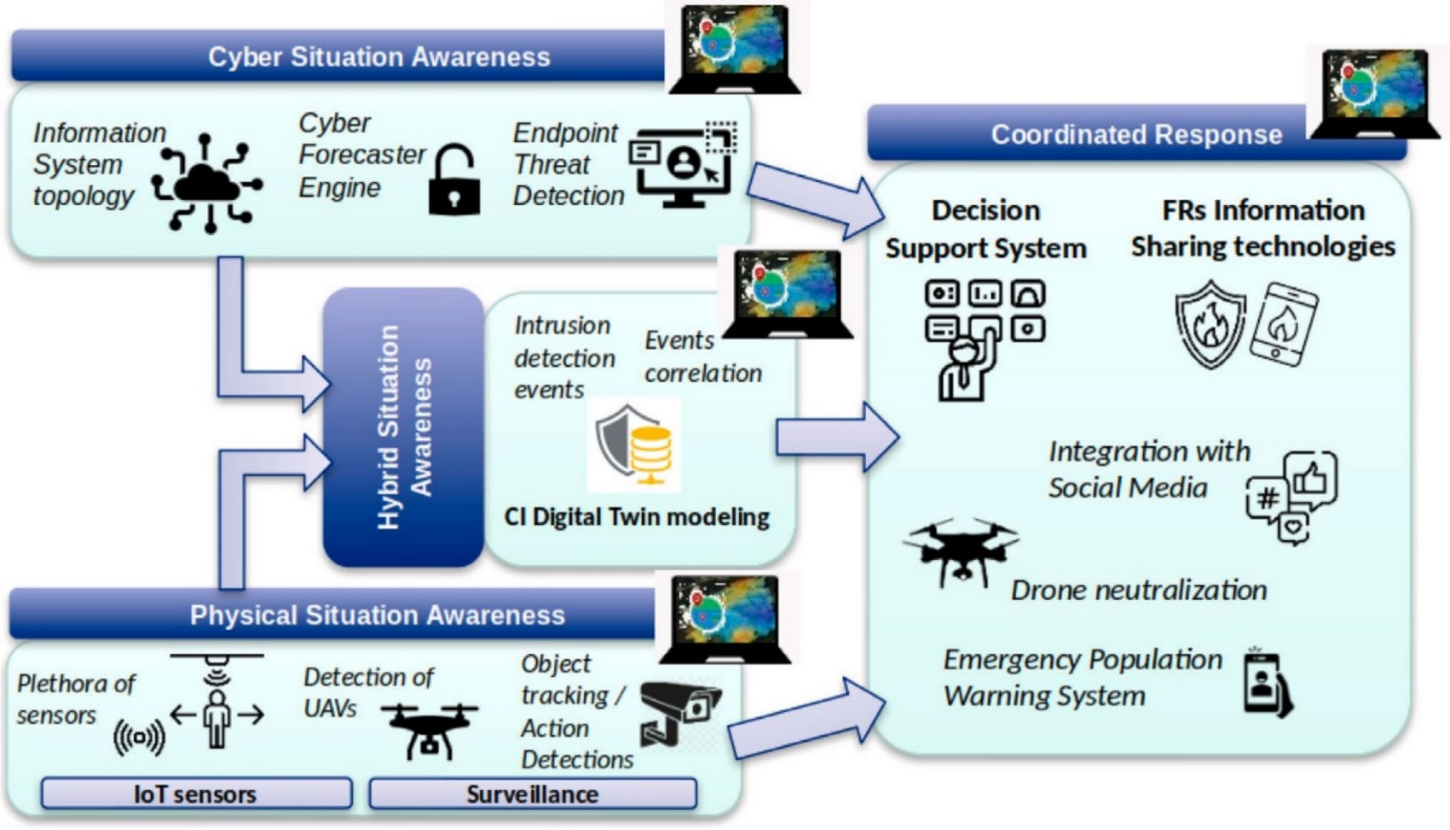
The elements of the PRAETORIAN platform, according to Muñoz-Navarro et al. (2025)

The project provides a toolset of (i) a physical situation awareness system, (ii) a cyber situation awareness system; (iii) a hybrid situation awareness system, which includes digital twins of the infrastructure under protection; and (iv) a coordinated response system. The schematics of the relations/interactions between these elements is depicted in Fig. 1 (Muñoz-Navarro et al. 2025). Indications from the cyber and physical security awareness systems—both triggered by a plethora of different specialised sensors—are accumulated in the hybrid situation awareness system, which then correlates the events by utilising digital twin architectures of the critical infrastructures. All three awareness systems are interconnected with the coordinated response, which provides decision support based on information received and gives action advice to first responders, population, and other stakeholders.

The PRAETORIAN toolset aims to assist security managers of CIs by providing decision-making support and enabling to anticipate and withstand potential security threats, whether they are cyber, physical, or a combination. It is intended to support operators to protect their own as well as linked CIs, as any disruption may have a significant impact on operational efficiency and overall safety of the surrounding population.

The project specifically handles man-made cyber and physical attacks affecting linked CIs by fostering prevention, detection, and—in case of an ongoing attack—mitigation of the attack. PRAETORIAN also addresses cascading effects on normal operations in linked CIs by aiming to increase the resilience of these connected CIs. To this end, the system predicts cascading effects, proposes a unified response among CIs, and assists first responder (FR) teams.

In order to evaluate the operational feasibility of the overall toolset and the underlying operational concept, validation exercises were conducted to obtain expert feedback. The validation exercises were conducted based on the European Operational Concept Validation Methodology (E-OCVM; EUROCONTROL 2010), a framework originating from ATM research. This paper presents selected results from the PRAETORIAN validation exercises.

Four validation scenarios that contain a wide range of attacks and CIs were developed within the project. There was one validation exercise per scenario in which the respective scenario was presented to a selected group of participants. Based on the maturity level of the system, the validation approach combined presentations, and elements of a cognitive walkthrough with selective hands-on-phases. Since PRAETORIAN is aimed to be generalisable to different settings and not tailored to one specific scenario, the results of the four validation exercises will be reported in an aggregated manner and not scenario specific, covering the entirety of attacks and CIs in this work. Further, only the assessed results regarding the overall system will be considered, i.e., the recorded results concerning the individual tools will not be included.

The chosen validation approach was already described and discussed in the Stelkens-Kobsch et al. (2023) work and parts of the results presented in a condensed approach by Piekert et al. (2025). This includes, e.g., a discussion of the suitability of the chosen validation approach, lessons learned from the validation exercises, and an overall assessment of all validation objectives. This work will extend the previous publications by provision of additional details and previously omitted elements.

## Methods

The data was analysed in regard to pre-defined objectives and acceptance criteria (AC). These will be explained in more detail in the following two subsections. The objectives and the criteria were jointly defined within the aforementioned project PRAETORIAN and with its project partners.

### Objectives

Five distinct objectives were defined to more suitably group the different aspects that needed to be answered for this validation. These objectives are provided in Table 1 with their section and titles.

**Table 1:** Objective sections and titles

Objective section	Objective
A	Better understanding of attacks and consequences
B	Better resilience and improved coordinated response
C	Usability and acceptance of solution
D	Information provision to the public
E	Cost–benefit aspects

Objective section A groups those aspects that allow to assess if the operators that use the proposed system gain a better understanding of the attacks and the potential consequences stemming from these.

Section B answers aspects that indicate an improved coordinated response against those attacks and if better resilience can be achieved, based on the information gained from the system.

Section C provides insight into usability and acceptance aspects provided by the different operators by their subjective perception.

Objective section D addresses the information provision to the public during events handled with the solution.

Lastly, objective section E gathers elements that are concerned with cost–benefit aspects linked to the real-world deployment and system utilisation.

### Acceptance criteria

Seventeen acceptance criteria (AC) were evaluated using questionnaires and debriefing feedback. Acceptance criteria form the basis for the later decision if an objective has been achieved or how it is evaluated, based on the gathered data.

The subsets of the acceptance criteria provided in Table 2 allow to assess if the system helped to improve the understanding of any physical or cyber threats and their consequences in the interdependent network of critical infrastructure that were modelled in the overall systems.

**Table 2:** Acceptance criteria for objective A Better understanding of attacks and consequences

AC	The PRAETORIAN solution…
A1	… enhances situation awareness
A2	… enables a faster detection of cyber and physical threats
A3	… does not induce operator overload
A4	… provides the relevant information
A5	… provides helpful decision support

The criteria given in Table 3 for objective B allow to assess if it is possible to improve the resilience of the CIs, their neighbouring population, and environment and enable a coordinated response to an attack.

**Table 3:** Acceptance criteria for objective B Better resilience and improved coordinated response

AC	The PRAETORIAN solution…
B1	… enables a faster coordinated response to cyber and physical threats
B2	… improves the resilience of CIs
B3	… enhances teamwork between the parties involved, e.g., operators and first responders

Table 4 covers the human factor (HF)-related acceptance criteria and allow to answer if the overall PRAETORIAN toolset as well as its individual tools and functionalities is accepted and easy to use by the participants of the validation exercises.

**Table 4:** Acceptance criteria for objective C Usability and acceptance of solution

AC	The PRAETORIAN solution…
C1	… is accepted
C2	… is trustworthy
C3	… is usable
C4	… is intuitive to use
C5	… conforms to operators’ mental models

The single acceptance criterion listed in Table 5 addresses the aspect to share pertinent information on the risks associated to an event and the emergency response actions planned to overcome the incident with the public.

**Table 5:** Acceptance criteria for objective D Information provision to the public

AC	The PRAETORIAN solution…
D1	… allows faster sharing of relevant information with the public

Table 6 addresses ACs from a cost–benefit perspective. It helps to answer if the project results, regarding in real contexts of interdependent CIs, help to improve its overall efficiency, the cost-effectiveness, and if these provide societal benefit.

**Table 6:** Acceptance criteria for objective E Cost–benefit aspects

AC	The PRAETORIAN solution…
E1	… is efficient
E2	… is cost-effective
E3	… has societal benefit

### Sample

The overall number of participants was 24, divided into four exercises: there were five participants in exercise #1, eight participants in exercise #2, six participants in exercise #3, and five participants in exercise #4.

Due to the small sample size and data protection concerns, no information about age and gender was collected.

The participants were staff members from organisations included in the validation scenarios, but they were naïve about the contents of the scenarios. Among these were three first responder (FR) organisations, a laboratory, two hospitals, two ports, two airports, a power plant, and a hydropower plant. The participants provided written informed consent and received no additional financial compensation for their participation in the exercises.

Since each participant took on one or several defined roles in the validation scenarios, their work-related responsibilities were considered during the recruitment phase in order to create a match with their scenario role to the possible extent.

### Validation scenarios

Four validation scenarios demonstrated the PRAETORIAN solution’s functionalities in different situations. Each validation scenario contained cyber-, physical, or combined cyber-physical attacks. These were affecting multiple CIs, either directly or indirectly due to their cascading effects. Highly recognised subject matter experts, CI operators, and CI security researchers were involved in the scenario development. The scenarios are based on their experience and realistic estimations of possible attack scenarios.

#### Scenario #1

Scenario #1 presents a cyber-physical attack on a hydropower plant causing blackout and flooding, cascading to a hospital. Table 7 shows a high-level description of the course of action in this scenario. It is adapted from unpublished project material (Hingant et al. 2023).

**Table 7:** High-level information provided for the scenario 1, adapted from Hingant et al. (2023)

Step	Description
1	Due to the COVID-19 pandemic situation, the energy demand is lower than its usual level. HydroPowerPlant (HPP), one of the main energy production sites, is storing an enormous amount of energy generated by other power plants
2	HPP security centre is alerted by national authorities about potential cyberattacks targeting national Critical Infrastructures, which include that specific one
3	A Counter Unmanned Aerial Vehicle (C-UAV) system is temporary contracted and deployed at the power plant with the objective of reinforcing the physical security of the whole HPP facility
4	An experienced terrorist group, with the main purpose to completely unbalance the national energy network, finally decides to take action by taking advantage of the information (publicly available at the HPP official social media) of the extremely high amount of energy being stored at the power production site
5	After having shared CI-related sensitive information on his social media accounts in the previous weeks, some of the terrorist members have already gathered the HPP security administrator’s credentials
6	The terrorist team send aerial drones to the HPP site in order to collect aerial pictures of all the CI facilities
7	The C-UAV analysis centre, by means of the deployed C-UAV system, raises an alert to the HPP security centre after detecting the flight of the rogue drones over the CI environment
8	The hydropower plant decides to reinforce its physical security. To do so, two measures are taken: 1. The amount of HPP security members is increased 2. A local police force is alerted and stays in standby ready to act
9	The cyberterrorists finally manage to take remote full control of the hydropower plant’s access control and CCTV systems
10	Once the webserver managing the video surveillance cameras in charge of controlling the HPP entrances is disabled, a van of the terrorist group succeeds in easily accessing the CI facilities
11	The video analytics capability, which relies on video surveillance cameras managed by a different server than the hacked one hosting the CI’s main CCTV system, detects the van as an unknown and unauthorised vehicle
12	Thanks to the alert sent to the HPP security centre, both police force and all the security staff are alerted about the physical intrusion. The police staff joins the CI’s security members in few minutes
13	A second cyberattack is at this point achieved permitting to the terrorists to gain now full remote access to the HPP’s turbine control system
14	By the exploitation of a publicly available Industrial Control System (ICS) vulnerability in social media, the power production site’s turbines are configured to produce energy at their full capacity and release both the previously stored and the new generated one into the main energy grid
15	The HPP’s Security Operations Center (SOC) is finally alerted by the Cyber Security Alert (CSA) system about some unusual security-related cyber event patterns detected in the network of the power plant
16	Several cascading effects are forecasted as a result of propagating the events and threats detected both in the cyber and in the physical domains of the CI. One such effect threatens to turn the downstream hospital inoperational due to flooding from the nearby river and additionally by possible power outages
17	HPP security personnel in coordination with competent authorities, according to Decision Support System (DCS) recommendations, decide to urgently inform through the Emergency Population Warning System (EPWS) to all concerns, in particular, to other CIs in order to rapidly switch to their backup power systems

#### Scenario #2

Scenario #2 is about cyber-physical attacks on a power plant and a port. Table 8 shows a high-level description of the course of action in this scenario. It is adapted from unpublished project material (Hingant et al. 2023).

**Table 8:** High-level information provided for the scenario 2, adapted from Hingant et al. (2023)

Step	Description
1	A coordinated group of terrorists is looking to destabilise the national energy supply by a combined attack to some of the national CIs in order to both destabilise one of the oil supply chains and disrupt the electricity supply in the region
2	Hackers of the terrorist group have previously detected a vulnerability in the port operator’s (PO) VPN network which is due to a lack of software security updates. This VPN is responsible, among others, for ensuring the transmission of tidal information operated by the tidal levels’ announcement services
3	In the meantime, a corrupted employee of the power plant, acting as an insider, collaborates with the criminal organisation by connecting a malware USB key targeting the access control systems of the CI
4	The terrorist group finally manages to exploit the identified PO’s VPN vulnerability (by modifying the network packets’ labels) and disable the vessel traffic system of the adjacent port. The cyber forecaster engine (CFE) detects both the unavailability of the radar service and the incorrectness of the provided tide level information and sends the corresponding alerts
5	In the meantime, aerial drones loaded with explosives and targeting the PO’s oil terminal are detected by the C-UAV system, which triggers the corresponding alert to the port security team
6	By achieving propagation calculations with the information about these two simultaneous events at PO’s facilities with some additional suspicious activities aiming at CIs in the region and detected by the National Cybersecurity Agency (NCSA), a potential attack against the port and related CIs is inferred
7	The Decision Support System orchestrates emergency plans by alerting the local authorities (maritime and the regional officials), who activate the regional emergency operation centre (REOC)
8	At the power plant, a short time after, the CFE alerts the CI’s security team of a cyberattack against the access control system that prevents anyone from accessing its facilities. Consequently, the security office activates its corresponding emergency operation centre
9	The power plant site and the port take immediately measures to reinforce their property security: • On the one hand, additional security members are intended to strengthen both CI’s security • On the other hand, and to face the lack of aerial intrusion detection, the port’s C-UAV system is set to expand its viewing and detection area to the entire site
10	In the meantime, two clusters of explosive-loaded drones are driven to both attacked CIs: • 10 aerial drones are sent to the power plant and the PO’s oil terminal • 10 underwater drones are driven towards the fuel area of port
11	The C-UAV system identifies the aerial drones as hostile ones and detects their explosive load
12	The drone neutralisation system is immediately activated and neutralises all the aerial rogue drones at both CI facilities by using techniques such as, i.e., radio jamming or camera blinding lasers. However, for safety reasons the power plant has to be shut down to allow forensic investigations about the attack
13	A new alert is generated at PO’s security centre when the underwater drone detection system recognises the maritime unknown drones in the sea approaching the CI facilities
14	Even though PO’s port security staff manages to disable most of them, some of the underwater drones succeed in hitting one of the oil tanks, which explodes, burning the dock and starts spilling tonnes of fuel in the ocean
15	After REOC’s activation, the government official representative orders both Law Enforcement Agencies (local police, national police) and rescue services to coordinate the rescue operations at the affected CIs
16	With the objective of creating chaos and confusion, the criminal organisation disseminates false information in social media about national petrol supplies and reserves, which leads to a panic run of the citizens to the pumps and blocking the oil depots
17	The government official representative orders to activate the Emergency Population Warning System (EPWS) in order to alert the population about the situation. Simultaneously, the regional relief chain elevates to the national level. The national authority requests, within the framework of the European civil security mechanism, the sending of units specialised in hydrocarbon risks
18	The nearest hospital, which suffered a huge fire a few days before and that provoked its power backup system to be out of order, is quickly overloaded when receiving the injured people due to the lack of electricity. The adjacent airport, for its part, gets also congested by the collision of planes due to the smoke screen provoked by the oil tank explosion and the impossibility of landing. Moreover, the power plant’s shutdown leads to an important imbalance of the National power production at a time when electricity supply has to be provided to neighbouring countries

#### Scenario #3

In scenario #3, a cyber-physical attack on a port has cascading effects on a hospital and an airport. Table 9 shows a high-level description of the course of action in this scenario. It is adapted from unpublished project material (Hingant et al. 2023).

**Table 9:** High-level information provided for the scenario 3, adapted from Hingant et al. (2023)

Step	Description
1	The port is alerted by EU security officials and authorities about potential (cyber and/or physical) terrorist attacks against some European Critical Infrastructures
2	The port immediately takes measures to raise both its physical and its cyber counter intrusion security: • On the one hand, security staff is increased and a C-UAV system is temporary deployed as a complement to the current radar system • On the other hand, the network intrusion detection system is reinforced and the well-functioning of the server managing the port CCTV system is consistently verified
3	In the weeks prior to an attack to the port, an organised terrorist group succeed in gathering a confidential report about its IT systems vulnerabilities from social media
4	In the preparation of this attack, photos of the CI facilities that reveal physical security vulnerabilities are collected by the terrorists using an aerial drone
5	The flight of the aerial drone is detected by the C-UAV system, which alerts the port security centre. This one, at its turn, also informs the nearby airport about this intrusion
6	The port’s Security Operations Centre (SOC) informs the CI cybersecurity committee that some attempts of cyberattack against its IT systems have been detected. The preventive measures previously performed allow to partially deactivate this specific cyber threat
7	A crisis management plan is however immediately activated by the port security centre. As a result of this, both local police and extra security members are requested to go to specific CI locations to ensure the surveillance of these areas
8	At the same time, a different rogue drone is detected in the surroundings of the airport. The alarm raised by their own detection system, in combination with the previously alert received from the port, leads the airport to move to declare an extreme warning situation
9	Thanks to the exploitation of one of the IT vulnerabilities included in the security report previously obtained, some hackers of the terrorist group finally succeed in taking remotely control of the whole port access control system and disable it
10	Taking advantage of this lack of security at the port entrances, an explosive-loaded van of the terrorists accesses the CI facilities and goes directly to the fuel tank area, which is located near a docked cruise ship with more than 5000 passengers on board
11	This physical intrusion is however detected by two different physical security capabilities: • firstly, by the identification of the van plate as an unauthorised one; and • secondly, by the recognition of violent behaviours Both systems send a corresponding alert to the port security centre
12	Few seconds later, a powerful dirty bomb is detected inside the terrorist van. An alert is sent to the port security office, which informs immediately the hospital of the nearby city. Its emergency action plan is then activated in advance
13	A port security team is requested to urgently reach the oil tanks area. Nevertheless, when the terrorists notice the security staff approaching, the van is driven against the fuel tank and the bomb carried in it is remotely detonated
14	The enormous explosion is detected by a sound sensor located at the area and a new alert is transmitted to the port security centre
15	At the meantime, the SOC of the port also detects the cyber intrusion into the CI’s access control system and alerts the cyber security operators
16	By carrying out the propagation calculations with the information related to both the physical and the cyber detected events at the port, some cascading effects showing the consequences of the ongoing coordinated attack are estimated
17	As a consequence, both the airport and the city’s main hospital, as port related-CIs, are immediately informed about the terrorist attack at the port facilities. In addition, first responders and other rescue services are requested to take care of the victims of the explosion
18	Emergency messages are sent to the people geo-located at the port area to facilitate the evacuation tasks of the CI’s facilities
19	The airport, which already was in an extreme warning mode, decides to finally and emergently lockdown all its facilities and interrupt all the ongoing activities
20	The city’s main hospital, coinciding with a temporary overload situation due to the COVID-19 pandemic, receives a huge number of victims of the explosion, mainly passengers of the docked cruise, in the following hours and rapidly collapses

#### Scenario #4

Scenario #4 involves a cyber-physical attack on a laboratory proceeding cross-border to an airport. Table 10 shows a high-level description of the course of action in this scenario. It is adapted from unpublished project material (Boumann et al. 2023; Hingant et al. 2023).

**Table 10:** High-level information provided for the scenario 4, adapted from Boumann et al. (2023) and Hingant et al. (2023)

Step	Description
1	A city-based bio security level 3 laboratory in nation A is targeted by a group of terrorists. On the dark web, they find the blueprints of the building in which the laboratory is situated. A relative of one of the employees of the laboratory is approached by the terrorists. He is blackmailed and forced to steal and make a copy of the employee’s ID card as well as to find out a 4-digit code used at the laboratory. With the necessary information the terrorists wait for the best moment to attack
2	Terrorists approach a corrupted airport employee who is given instructions on how to disable the airport check-in system. It is summer and numerous tourists are coming to this nation B. Nation B’s airport is the busiest airport in the region. The airport is a 2-h car drive away from the city where the laboratory is located. At that time, many tourists from central European countries are driving by car to nation B to take a vacation at the Mediterranean
3	The terrorist uses previously obtained ID card to enter a changing cabin of the laboratory
4	The terrorist does not perform the procedure specific for BSL-3 laboratory when in the changing cabin. PRAETORIAN video analysis can detect that “something strange” is happening; the analysis can detect a person did not take enough time to prepare at the changing cabin, but due to the strict procedures it takes time to watch those video recordings and confirm the primary suspicion
5	The terrorist uses previously obtained 4-digit code to enter the main laboratory room
6	The terrorist opens a fridge and steals the sample and the terrorist inserts a USB with malware into the laboratory PC and exits the laboratory
7	The terrorist splits the sample into two parts, one for himself and the other for his teammate. The terrorist takes a car and drives towards the city near nation B’s airport. The teammate takes another car with the drone inside and also drives towards the city near nation B’s airport, but choosing other roads
8	PRAETORIAN’s CSA detects the cyber intrusion, but it takes time to realise that a sample is stolen and to analyse which sample is missing. Analysing the video confirms an unauthorised access to the laboratory. Nation A’s authorities are alerted, but the information did not cross over to nation B
9	The malware is launched from the USB after a first legitimate user is logged in to the laboratory PC
10	The laboratory crew performs a forensic analysis of the event to decide on the next steps
11	An alert is issued to related CIs
12	In preparation of the attacks, the adversaries engaged a corrupted employee to physically disable the check-in process. The cyber forecaster engine detected the vulnerability exploitation and alerted the IT staff. However, a group of hackers immediately managed to penetrate the network and installed the WannaCry ransomware on flight information display system (FIDS) and on the Common Used Terminal Equipment (CUTE) platform for passenger handling. The infected FIDS system enables the attackers to present messages on the displays in the terminal building, and the infected CUTE platform disables the check-in process, which causes people to gather at the check-in area and creates a crowded environment
13	Both attackers approach the airport with the aim to use the bioweapon in the indoor and outdoor area of the airport. Their goal is to have a simultaneous attack inside and outside
14	Attacker 1 enters the airport and moves around near the check-in counters while waiting for his teammate to fly the drone to the targeted area
15	Attacker 2 drives to a location near the airport where he has visibility of the location he is targeting with the drone armed with bioweapon. Then he flies the drone towards the airport. This situation is detected by the C-UAV and alerts physical security officers at the airport, and they are engaged in the dealing with this attack. Thanks to the C-UAV, the drone is neutralised at an inaccessible area near the airport. The remains of the UAV and the cargo it was carrying are scattered in a nearby forest
16	The PRAETORIAN platform uses all the previously gathered information to provide a coordinated response. The actions then result in the apprehension of the attackers by police and security forces

The validation scenarios were presented using presentation slides supported by narrations, pictures, or videos. An initial overview of the scenario was provided, followed by a four-step approach:

- First, each scenario step was narrated in more detail.
- Second, participants were asked about their current procedures and operations, e.g., which systems and procedures would currently be used in such situations.
- Third, the relevant PRAETORIAN tools for the scenario step were demonstrated by showing the human–machine interfaces (HMIs) via screen sharing. Alerts were simulated in the background. Since participants had received role-dependent credentials for the different tools, they were able to interact with the HMIs along on their own screens. Some participants were asked to share their own screens and then instructed on using the HMIs, while in most other cases, developers demonstrated the tools by sharing their own screens. It became apparent that an interactive approach is advisable to obtain more valid feedback by the exercise participants.
- Fourth, participants’ feedback on the demonstrated tools was gathered.

The described four-step approach was utilised for most scenario steps, but deviations were possible depending on the course of the scenario. It should generally be noted that the presentation slides, including the tools shown and the questions asked, were tailored specifically for each of the four validation scenarios. Further, scenario #2 deviated in the third step as some scenario steps were executed live on a digital twin.

An in-depth description of the four scenarios is available as a project report on the European Commission’s Server (Laguna et al. 2023).

### Questionnaires

Three different types of questionnaires were used for the assessment done by the operators. In principle, these were similar for all the participants; however, exercise-specific adapted formulations were incorporated.

#### Debriefing questions

After each validation scenario, a debriefing was conducted with all present participants. The debriefing questions were identical for all validation exercises. Participants were asked what benefits they see in PRAETORIAN’s concept and technology and how this concept and technology could be improved.

It was subsequently pointed out to the participants that PRAETORIAN can share all information received during the previously presented attacks with other CIs on a European scale. The participants were asked what benefits and what obstacles they see in this kind of cooperation. Lastly, they were asked for final comments.

#### Bespoke validation questionnaire

A bespoke validation questionnaire was created on the Open Source survey solution LimeSurvey (LimeSurvey GmbH 2025) which participants received after the validation exercise. It contained questions and statements about the validation scenario, the overall PRAETORIAN solution, and its individual tools.

Each solution-related item was mapped to an acceptance criterion. This work will present results from 31 items related to the selected objectives in Table 1 and ACs in Tables 2, 3, 4, 5, and 6 that focus on the overall PRAETORIAN solution.

This comprises 31 five-level Likert items rated from 1 (strongly disagree) to 5 (strongly agree). Means (*M*) and standard deviations (*SD*) were calculated for each item. The neutral rating of 3 (neither agree nor disagree) served as a cut-off criterion, i.e., to fulfil an AC, related statements had to be rated with a mean rating of 3 at the minimum. In the case of inverse statements, mean ratings had to be below 3. Free text answers to open questions (independent or follow-up questions based on participants’ agreement or disagreement with specific statements) will not be reported in detail, but may serve to further elaborate on quantitative results in the discussion if relevant.

Results from items focusing on individual tools or the validation scenarios themselves are not reported in this work.

#### System usability scale

The System Usability Scale (SUS; Brooke 1995) was administered after each validation exercise along with the bespoke validation questionnaire. The SUS was used for the evaluation of the PRAETORIAN solution’s usability in the context of AC C3 “The PRAETORIAN solution is usable”.

It comprises 10 five-level Likert items ranging from 1 (strongly disagree) to 5 (strongly agree), from which a SUS score between 0 and 100 is calculated. *M* and *SD* were calculated from the SUS scores and interpreted following Bangor et al. (2009) and Brooke (2013).

### Procedure

The validation exercises were performed remotely using web-conference tools. Each validation scenario was run once, so four validation exercises were conducted in total. Because the validation scenarios were specific to the involved CIs and FR organisations, each validation exercise was attended by only one group of participants. In the sense of an iterative process, insights from conducted validation exercises were used to improve the subsequently following validation exercises, e.g., regarding the selection of participants, procedure, or implementation of tools.

Each validation exercise lasted one working day. In the morning, informed consent was collected from the participants, and they received project information and an introduction to the PRAETORIAN tools. The morning ended with questions and answers, and participants were encouraged to ask questions as needed during the exercise.

Participants received specific credentials for their exercise roles and logged in to the PRAETORIAN tools.

In the afternoon, the validation scenario was presented. During this, participants were asked for their feedback, and afterwards, the debriefing was conducted with all participants of the exercise. Online questionnaires were completed either immediately or later, depending on individual time constraints.

The validation exercise of scenario #2 deviated partially. Due to events outside the responsibility of the project, some participant companies were subject to industrial action and hence not all participants could join the exercise live at the previously scheduled day. As mitigation it was decided that this exercise was going to be recorded for the absent participants and that they would sent their written feedback afterwards on the following day.

### Data analysis

Quantitative data from the questionnaires were analysed descriptively using IBM SPSS Statistics 26 (IBM Corp. 2019). *M* and *SD* for the selected bespoke questionnaire items and SUS scores were calculated over all scenarios for the 24 participants.

Free text answers to open questions from the bespoke questionnaire were categorised, but these will not be reported in this work in detail. However, they will be used to elaborate on certain discussion points in the next section. These results were analysed with regards to their assigned AC.

Debriefing feedback was analysed in an aggregated manner over all scenarios and categorised by AC or, if there was no fitting AC, identified as additional feedback. Feedback from the validation scenario playouts was used to gather lessons learned for future research.

## Results and discussion

The results from the bespoke validation questionnaire and the SUS are reported and discussed in the following subsections. They are grouped in the context of their corresponding objectives and acceptance criteria to provide better coherent readability. In addition to the statistical evaluation, verbal and textual responses gathered in the questionnaires and debriefings will be regarded, and limitations of the validation exercises will be considered.

It must be noted that it is not possible to include all data collected during the validation exercises in the scope of this work, partially due to privacy and confidentiality considerations. Based on selected results, a more global evaluation that does not consider all feedback in detail is therefore provided.

Section 4 explains the ACs A1–A5 under objective A “Better understanding of attacks and consequences”, Sect. 5 provides ACs B1–B3 from objective B “Better resilience and improved coordinated response”, Sect. 6 reports ACs C1–C5 from objective C “Usability and acceptance of solution”, Sect. 7 covers AC D1 from objective D “Information provision to the public”, and Sect. 8 addresses ACs E1–E3 from objective E “Cost–benefit aspects”. Following the provision of the results, these are discussed in the same sections.

The table headings for Tables 11, 12, 13, 15, and 16 provide means denoted by *M* and the computed standard deviations denoted by *SD* for the 31 bespoke questionnaire items. The final column shows if the cut-off criterion was reached, i.e., if the mean was at least equal to or higher than 3, which is the neutral threshold. For inversely phrased statements (indicated additionally by the letter b), the cut-off criterion is reversed. Passing of the cut-off criterion or not is indicated by either a (green) check mark √ or a (red) ×.

**Table 11:** Results of the bespoke questionnaire items (all *N* = 24) for objective A

Acceptance criterion	Item	*M*	*SD*	√/×a
***Objective A: Better understanding of attacks and consequences***				
A1. Situation awareness	1. Compared to the current situation, I think the PRAETORIAN system will enhance my situation awareness	4.13	0.45	√
	2. The PRAETORIAN system helps to obtain a complete picture of the situation	4.25	0.61	√
A2. Faster detection of threats	3. Compared to the current situation, I think the PRAETORIAN system will enable a faster detection of cyber and/or physical threats	3.92	0.83	√
A3. No operator overload	4. The PRAETORIAN system displays too much information	3.13	0.95	×b
A4. Relevant information	5. The PRAETORIAN system provides the information that I need	3.58	1.02	√
	6. The interfaces used to share data with external sources and organisations provide the right information	3.54	0.83	√
A5. Helpful decision support	7. The PRAETORIAN system provides helpful decision support	4.04	0.62	√

a *√* means: the cut-off criterion was reached (*M* ≥ 3); *×* means: the cut-off criterion was not reached (*M* < 3), b Inverted item (cut-off criterion reversed

**Table 12:** Results of the bespoke questionnaire items (all *N* = 24) for objective B

Acceptance criterion	Item	*M*	*SD*	√/×a
***Objective B: Better resilience and improved coordinated response***				
B1. Faster coordinated response	8. Compared to the current situation, I think the PRAETORIAN system will enable a faster coordinated response to physical threats	3.71	1.00	√
	9. Compared to the current situation, I think the PRAETORIAN system will enable a faster coordinated response to cyber threats	3.92	0.72	√
	10. Compared to the current situation, I think the PRAETORIAN system will enable a faster coordinated response to combined physical and cyber threats	4.04	0.91	√
B2. Improved resilience	11. Compared to the current situation, I think the PRAETORIAN system will improve the resilience of Critical Infrastructures	3.83	0.70	√
B3. Enhanced teamwork	12. Compared to the current situation, I think the PRAETORIAN system will enhance communication between the parties involved, e. g. operators and first responders	3.88	0.80	√
	13. Compared to the current situation, I think the PRAETORIAN system will enhance coordination between the parties involved, e. g. operators and first responders	3.83	0.70	√

a *√* means: the cut-off criterion was reached (*M* ≥ 3); *×* means: the cut-off criterion was not reached (*M* < 3)

**Table 13:** Results of the bespoke questionnaire items (all *N* = 24) for objective C

Acceptance criterion	Item	*M*	*SD*	√/×a
***Objective C: Usability and acceptance of solution***				
C1. Acceptance	14. The PRAETORIAN system is compatible with procedures and systems currently used in operations	3.04	0.95	√
	15. I would like to use the PRAETORIAN system in real operations	3.88	0.85	√
	16. Compared to my current systems, the PRAETORIAN system provides advantages	4.00	0.51	√
	17. The PRAETORIAN system needs improvement.^b^	4.13	0.68	×b
	18. Compared to my current systems, the PRAETORIAN system provides innovations	4.17	0.70	√
C2. Trust	19. I think I would trust the information provided by the PRAETORIAN system	3.96	0.46	√
C3. Usability	20. The PRAETORIAN system is user-friendly	3.00	1.18	√
	21. The interfaces used to share data with external sources and organisations were easy to use	2.79	1.02	×
C4. Intuition	22. The PRAETORIAN system is intuitive to use	2.83	1.05	×
C5. Conformance with mental models	23. The PRAETORIAN system could be easily integrated in my current workflow	2.71	0.95	×
	24. The PRAETORIAN system is scalable, modular and flexible	3.58	0.88	√
	25. The PRAETORIAN system should raise warnings when sensors, critical process or any related modules are not available	4.46	0.51	√
	26. The PRAETORIAN system should offer a possibility to consult status and historical data	4.13	0.85	√
	27. The PRAETORIAN system conforms to my expectations	3.67	0.82	√
	28. The PRAETORIAN system conforms to my mental model of how the system should work	3.54	0.93	√

a *√* means: the cut-off criterion was reached (*M* ≥ 3); *×* means: the cut-off criterion was not reached (*M* < 3), b Inverted item (cut-off criterion reversed

Further, it is necessary to point out that some items of the provided ACs do not fully satisfy the equation |*M* − *SD*|> 3. The standard deviations for these cases exceed a value that can lead to a difference evaluation result where the element has a *M* value lower than the cut-off criterion 3. However, due to consideration of the participant feedback associated with these elements, in nearly all cases the elements and the ACs were considered to be fulfilled. This applies to the elements A3.4, A4.5, A4.6, B1.8, C1.14, C3.20, C5.24, C5.27, and C5.28. The only inverted element is C5.23, where the cut-off criterion is used in an inverted approach (see Table 13 in Sect. 6).

**Fig. 2:**
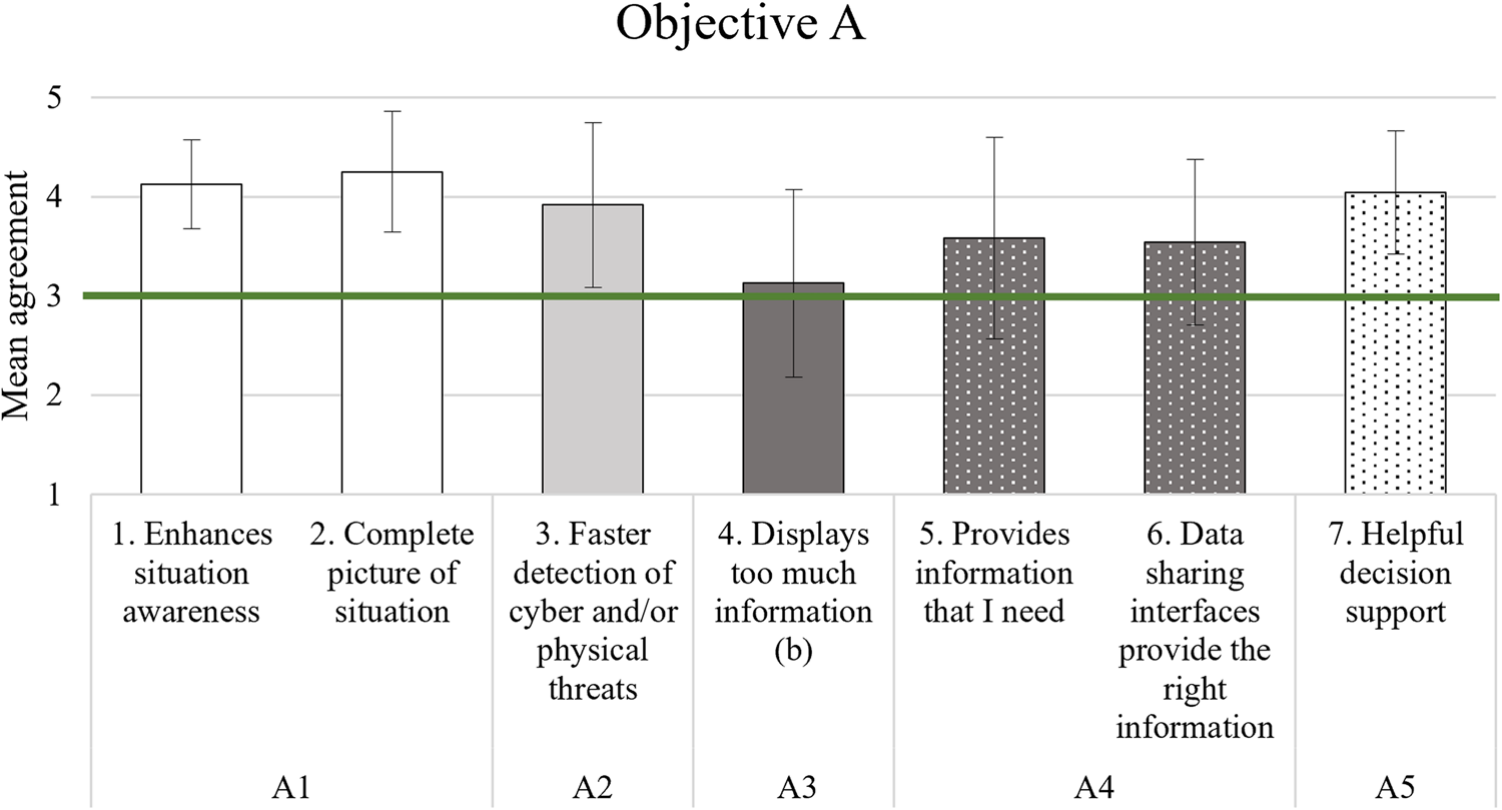
Graphic representation of objective A analysis results

**Fig. 3:**
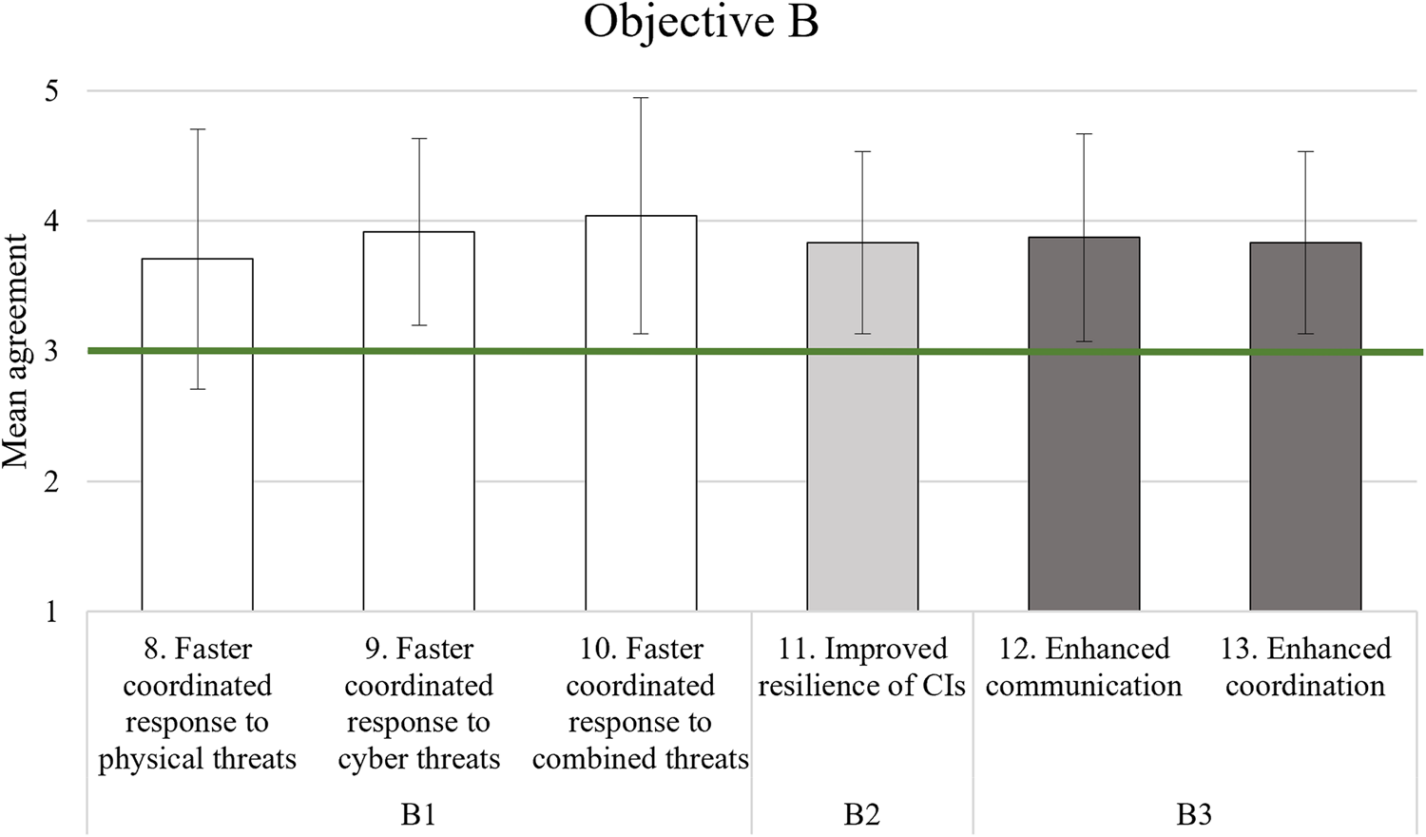
Graphic representation of objective B analysis results

**Fig. 4:**
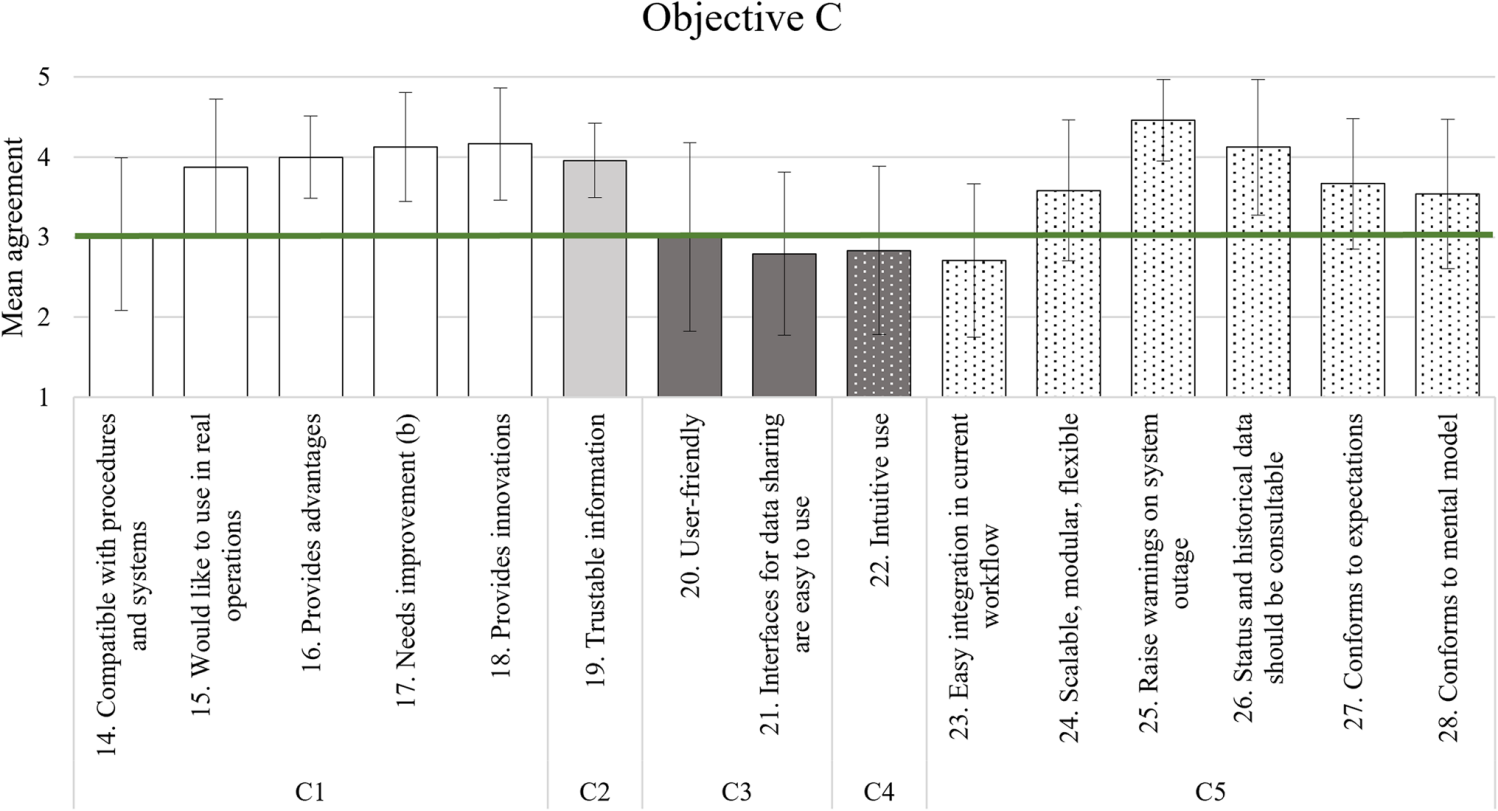
Graphic representation of objective C analysis results

**Fig. 5:**
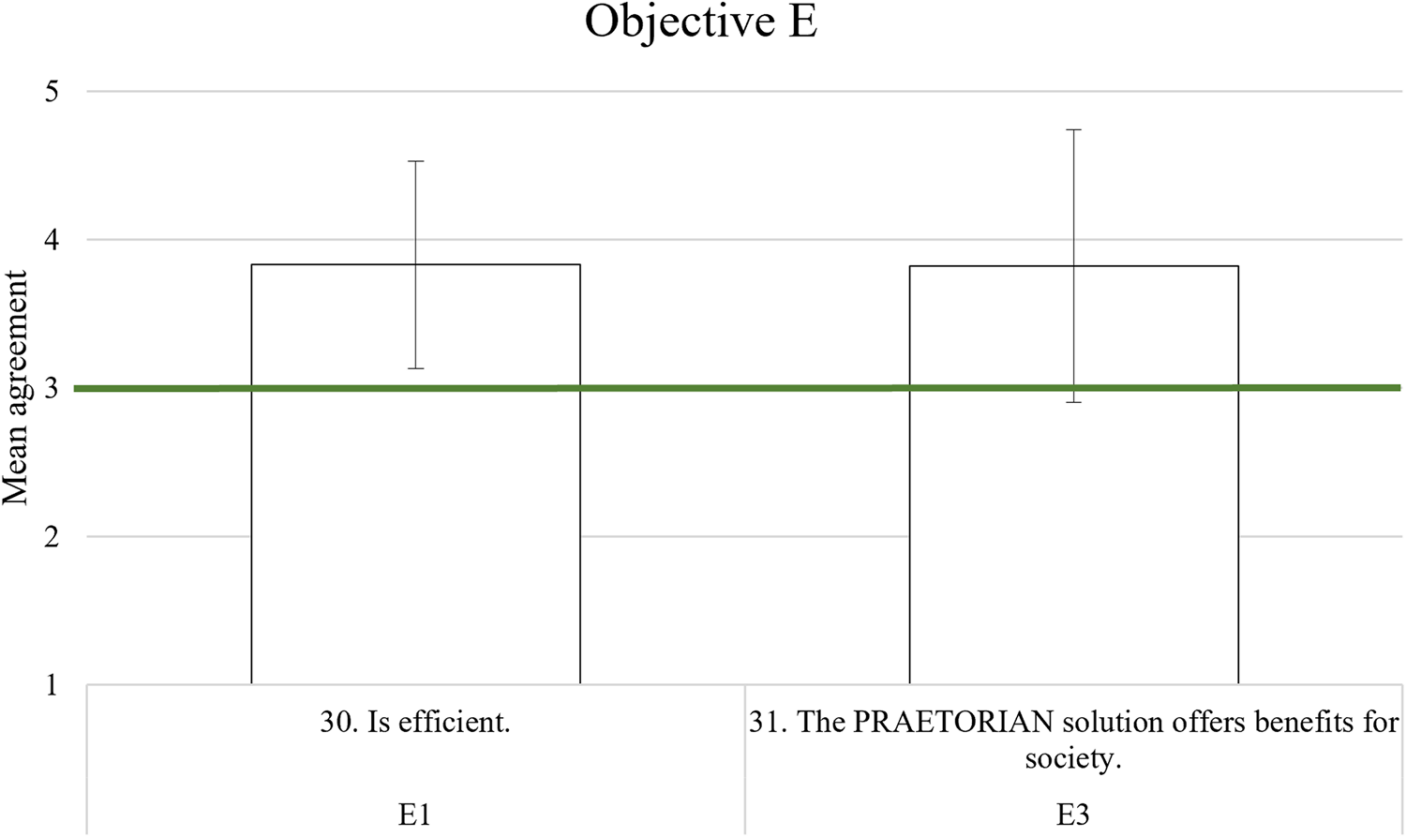
Graphic representation of objective E analysis results

For a quicker understanding of the results, especially regarding the cut-off criterion value of 3, Tables 11, 12, 13, and 16 have a graphical representation following the table. Each item of an AC is grouped in similar colour or pattern in the Figs. 2, 3, and 4, while Fig. 5 due to simplicity only has its two elements in the same colour/pattern.

### Better understanding of attacks and consequences

From Table 11 it can be seen that the results for the first objective were satisfying with regard to situation awareness, a faster detection of cyber and physical threats, the relevance of provided information, and the quality of decision support (ACs A1, A2, A4, and A5). Especially the enhanced situation awareness (items 1 and 2) and the quality of decision support (item 7) were rated positively.

However, in the context of AC A3 “The PRAETORIAN solution does not induce operator overload”, participants slightly agreed that the system displays too much information (item 4). In this case, the cut-off criterion was not met and the success criterion not achieved. Figure 2 depicts the results graphically.

Within this objective, the ACs regarding situation awareness (A1), a faster detection of cyber and physical threats (A2), the relevance of information (A4), and helpfulness of decision support (A5) were considered to be satisfying. The enhancement of situation awareness and the helpfulness of the decision support provided by the system were identified as benefits of the PRAETORIAN system in particular. Regarding the topic of situation awareness, some participants also named the integration of cyber and physical threats and the integration of safety and security issues as benefits during the debriefing. Regarding the relevance of information, some participants positively mentioned the availability of real-time information, sensor information, information about cascading effects, and about previous or on-going attacks at other CIs.

In the debriefings, participants expressed several ideas for additional features which could further improve the system in relation to the mentioned ACs. This included, e.g., additional types of sensors, a weather forecast, or a more extensive integration of individual CIs’ resources.

As the PRAETORIAN system seems to display too much information in some instances, it might cause operator overload. Therefore, AC A3 (no operator overload) cannot be considered fulfilled. As pointed out during one of the scenario playouts by participants, filtering options could help to mitigate this. However, the reported (slight) overload of operators could also be a result of a lack of training or familiarisation with the PRAETORIAN system, as participants did not receive training prior to the validation exercises. More extensive tool-related trainings could be an additional mitigation step. Considering this, the AC was evaluated to be partially satisfied.

### Better resilience and improved coordinated response

Table 12 shows that all items relating to the second objective (comprising B1, B2, and B3) were on average rated sufficiently high. Participants indicated slight agreement that the system could enable a faster coordinated response to cyber and physical threats (items 8 to 10), that it could improve the resilience of CIs (item 11) and that it could enhance teamwork between the involved parties (items 12 and 13). Figure 3 shows the corresponding graphical representation.

The results concerning the resilience and coordinated response to attacks were satisfying overall. This includes participants’ opinions about PRAETORIAN enabling a faster coordinated response to cyber and physical threats (B1), improving the resilience of CIs (B2) and enhancing teamwork between the involved parties (B3).

In the debriefings, the possibility of exchanging experiences and transferring knowledge between different actors was highlighted as a strength of the system multiple times, along with the ability to adjust or prepare responses to an incident. Positive remarks regarding communication, coordination, and information exchange were received. Nevertheless, participants also pointed out challenges, e.g., ensuring interoperability between CIs and establishing standardised conditions. Furthermore, it became apparent that more useful communication paths need to be constituted within the system. For example, some participants expressed that communication should run across a central communication point instead of direct inter-CI communication. As one lesson learned from the validation exercises, additional actors should be included in the system in order to reflect the communication workflows of end-users appropriately, e.g., authorities or additional groups of FR.

### Usability and acceptance of solution

The results for the third objective were heterogeneous, see Table 13. Regarding system acceptance (C1), the average ratings met the cut-off criterion except for item 17: on average, participants agreed that the PRAETORIAN system needs improvement. However, they also overall indicated, e.g., that the system provides advantages (item 16) and innovations (item 18). While mean ratings regarding the compatibility of PRAETORIAN with existing procedures and systems (item 14) passed the cut-off criterion, it should be noted that participants’ average agreement about this item was comparably low. Depicted in Fig. 4 is the graphical representation of the objective C results.

Overall, participants indicated sufficient trust in the information provided by the system (item 19, C2).

Concerning usability (C3), not all results met the cut-off criterion. Participants’ average agreement about the user-friendliness of the system was neutral (item 20) and therefore still sufficient. However, participants slightly disagreed that the interfaces used to share data with external sources and organisations are easy to use (item 21). Table 14 shows the *M* and *SD* for all scenarios and their overall mean SUS score, which was *M* = 53.22 (*SD* = 16.45), which corresponds to a usability slightly above “OK” according to Brooke (2013).

**Table 14:** Results of the System Usability Scale for objective C, AC C3

	***M***	*SD*
Scenario #1	58.00	4.81
Scenario #2	47.81	21.77
Scenario #3	64.58	11.45
Scenario #4	42.50	10.75
All scenarios	53.22	16.45

Furthermore, participants on average did not agree that the system is intuitive to use (item 22, C4).

In the context of the system’s conformance to participants’ mental models (C5), results for most items met the cut-off criterion. Among all items related to this AC, participants on average indicated the highest agreement that PRAETORIAN should raise warnings on system outage (item 25) and that there should be consultable status and historical data (item 26). However, on average they did not agree that PRAETORIAN could be easily integrated into their current workflows (item 23).

The third objective comprises ACs concerning acceptance (C1), trust (C2), usability (C3), intuition (C4), and conformance with operators’ mental models (C5). Acceptance of the system was evaluated to be satisfying overall. For example, participants agreed that PRAETORIAN would provide advantages and innovations compared to their current systems. When asked about the advantages provided by PRAETORIAN, participants named several advantages related to both the concept and the technology. This included, e.g., advantages related to teamwork, the integration of cyber and physical components, or receiving real-time information. Nevertheless, participants also indicated that improvements are needed. A large proportion of proposed improvements concerned usability-related aspects. PRAETORIAN’s compatibility with applied procedures and systems should be further investigated, seeing that participants expressed neutral attitudes in this regard. For example, participants voiced concerns that PRAETORIAN might be incompatible with procedures for sharing of confidential data, or General Data Protection Regulation. Furthermore, when asked which challenges they foresee in implementing PRAETORIAN, participants’ answers included, e.g., challenges related to the compatibility with systems, procedures and regulations already in place, cost of the system, ensuring security of PRAETORIAN itself, or willingness of companies to share data.

Trust in the system was rated to be satisfactory, though some participants stressed that it is essential to ensure the security and integrity of the PRAETORIAN system itself in real operations. The integration of a large amount of sensitive (and partially confidential) data introduces the risk of elevating the system to a single point of failure.

In particular, usability and intuitive use of the system were identified as areas for improvement, as participants did not find the overall system intuitive. Therefore, the results regarding intuition were considered unacceptable. This again highlights the need for extensive training. While in need of improvement, the usability of the overall system was still considered partially satisfying. The overall SUS score indicated low usability in a marginal, but not yet unacceptable range (Bangor et al. 2009; Brooke 2013) and the participants indicated an overall neutral attitude concerning the system’s user-friendliness. Some participants proposed that there should be a more consistent look and use between the different HMIs or less HMIs overall. Seeing that the PRAETORIAN system was not at a fully mature stage during the validation exercises, weaknesses regarding usability were to be expected and will likely be enhanced with increasing maturity level.

Concerning the system’s conformance with end-users’ mental models (i.e., in how far the system fulfils end-users’ expectations), the participants mostly expressed neutral to positive attitudes. Nevertheless, this AC was evaluated to be only partially satisfying. This is because the easy integration of the system in participants’ current workflows seems to need improvement.

### Information provision to the public

The results for the fourth objective was positive, see Table 15. The results from the bespoke questionnaire were satisfying. Participants’ agreement to PRAETORIAN allowing faster sharing of relevant information with the public was above medium with *M* = 3.54 (*SD* = 0.88). In the debriefing, some positive mentions of individual tools used for sharing information with the public were identified.

**Table 15:** Results of the bespoke questionnaire items (all *N* = 24) for objective D

Acceptance criterion	Item	*M*	*SD*	√/×a
***Objective D: Information provision to the public***				
D1. allows faster sharing of relevant information with the public	29. Compared to the current situation, I think the PRAETORIAN system will allow faster sharing of relevant information with the public	3.54	0.88	√

a √ means: the cut-off criterion was reached (M ≥ 3), × means: the cut-off criterion was not reached (M < 3)

The fourth objective was focussed on the inclusion of the general public by (faster) provision of suitable information about ongoing events. Results from the bespoke questionnaire were satisfying for AC D1. The participants’ agreement to PRAETORIAN allowing faster sharing of relevant information with the public was above medium. In the debriefing, some positive mentions of individual tools used for sharing information with the public were identified.

For context, it has to be considered that several participants reported not having sufficient permits for sharing information with the public, as responsibilities for this are allocated to other authorities. Possibly, validation participants were not able to draw a valid comparison between the current situation and the situation using PRAETORIAN due to this. It is recommended to investigate this further by also addressing end-users with the relevant responsibilities. However, depending on the different responsible authorities, it was suggested that access to the PRAETORIAN system should be made available for these entities to benefit from the consistency of the information in the system regarding ongoing events.

### Cost–benefit aspects

For the integrated PRAETORIAN system, results of the bespoke questionnaire statements regarding E1 “The PRAETORIAN solution is efficient” was given an acceptable overall mean rating of *M* = 3.83 (*SD* = 0.70), as is provided in Table 16. The AC E3 “The PRAETORIAN solution has societal benefit” was given an overall mean rating of *M* = 3.83 (*SD* = 0.92). Figure 5 shows the results graphically.

**Table 16:** Results of the bespoke questionnaire items (all *N* = 24) for objective E

Acceptance criterion	Item	*M*	*SD*	√/×a
***Objective E: Cost–benefit aspects***				
E1. is efficient	30. I think the use of the PRAETORIAN system is efficient	3.83	0.70	√
E3. has societal benefit	31. The PRAETORIAN solution offers benefits for society	3.83	0.92	√

a √ means: the cut-off criterion was reached (M ≥ 3), × means: the cut-off criterion was not reached (M < 3)

Regarding E2 “The PRAETORIAN solution is cost effective”, the reliable answering was not possible for the majority of the participants. In the validation preparation phase, it became obvious that the required participants needed to be operationally focussed and consequently were not involved in the accountant aspects required for answering cost–benefit-related questions. Therefore, there were no dedicated questions about PRAETORIAN’s cost-effectiveness in the bespoke questionnaire.

Objective E with its three ACs was evaluated only regarding the ACs E1 and E3, since E2 about cost-effectiveness was not addressed sufficiently due to the reasons already laid out above.

Acceptance criterion E1 results from the bespoke questionnaire were satisfying for the integrated PRAETORIAN system. However, results on individual tool level indicated that participants did not find clicking through the hybrid situation awareness system HMI easy and fast, indicating that the efficiency of its use could be improved and/or mitigated by sufficient training on the interfaces. No negative feedback regarding efficiency was identified in the debriefing. Two participants mentioned the integration of a large amount of information as a benefit of PRAETORIAN, which indicates the efficiency of the overall system. While the results are satisfying overall, it is recommended to evaluate the efficiency of the PRAETORIAN system further.

There were no dedicated questions about PRAETORIAN’s cost-effectiveness AC E2. Generally, it is recommended to conduct a cost–benefit analysis in order to evaluate cost-effectiveness. Although the project itself intended to conduct such an analysis, the results are not part of this work. Still, some feedback from the debriefing to be considered is reported. Cost of the system as well as (human) resources needed for its operation were identified as challenges by some participants. Furthermore, compatibility with CIs’ legacy systems and procedures should be ensured. Some participants guesstimated that the cost of PRAETORIAN will be too high, but that scalability of the system might positively influence cost-effectiveness.

Results from the bespoke questionnaire for the last AC E3 were satisfying. Participants’ agreement that the PRAETORIAN solution offers societal benefits was sufficient.

## Conclusions and recommendations for future work

Before drawing final conclusions on the validations, the accompanying limitations must be considered. The overall small sample size of *n* = 24 (answers) limits the explanatory power of the validation exercise results. This is especially true when looking at results on scenario level (ranging between *n* = 5 and *n* = 8 answers) or on tool level, i.e., results for tools that have been used only by a sub-group of participants.

Furthermore, it became apparent that the right target groups were not included in the validations in all cases. In some instances, participants reported not being the right user for a specific sub system. This was the case for one participant in the scenario #4 validation exercise because this participant did not have sufficient IT expertise to evaluate and use the CSA. For this reason, an effort was made in the other validation exercises to recruit participants with an expertise in IT. Still, in the scenario #1 validation exercise, one participant reported not being the right user for the PSA and CSA. In other instances, participants reported that in real-life environments, regulations foresee a different approach than implemented by the PRAETORIAN tool suite, and that other entities would be responsible for such actions. This included sharing of information on social media (IWSM) and issuing warnings to the population (EPWS). These circumstances may have limited them in their ability to evaluate the appropriate tools for these specific purposes.

Additionally, some limitations regarding the procedure of the exercises were identified. Firstly, due to unforeseen time constraints of some participants, those filled out the questionnaires after the validation exercises ended on their own. This means there was no opportunity for them to ask questions in case any statement was not clear. Further, a variable amount of time may have passed between the validation exercise being conducted and the participant filling out the questionnaires. This may have led to memory gaps or inaccurate recollection. To achieve a higher level of control, it would have been preferable for all participants to fill out the questionnaires directly after the scenario playout with the validation team aside if needed. Secondly, in the validation exercise of scenario #2, a group of participants could not be present due to a Union strike at their CI. Instead of participating live, these participants viewed recordings of the validation exercise and based their feedback on this recorded information. This means that participants’ understanding could not be checked, and they had no opportunity to ask questions. Nevertheless, the feedback provided by these participants seemed sufficiently high in quality and was therefore included in the data analysis.

Lastly, a concept-based approach was used for the validation exercises. For future stages of system development, it would be advisable to also run high fidelity HITL simulations, ensuring a higher level of realism and more opportunity to interact with the system in a coherent situation.

Therefore, as an overall conclusion, participants attributed several potential benefits to the PRAETORIAN system. The overall positive questionnaire results point towards operational feasibility in principle. The validation results must, of course, be interpreted within the context of the conducted validation exercises and their limitations. However, improvements on different levels are necessary in order to establish an overarching, holistic concept ready for implementation. This includes improvements regarding systems and HMIs, procedures, responsibilities, scalability considerations, and ultimately implementation, maintenance, and operational costs.

For example, to improve usability, the individual tools should be harmonised regarding their design and use. Furthermore, the validations showed that PRAETORIAN’s compatibility with end-users’ workflows, communication paths, currently used procedures, and systems needs to be considered. Following a holistic approach, PRAETORIAN is intended to be used by heterogenous CIs and end-users in Europe. To ensure compatibility with existing systems, procedures, workflows, and legislations is therefore a challenging endeavour, as harmonised standards often do not exist between national CIs, not to speak about cross-border connections. Some participants viewed standardisation as a necessary prerequisite for the implementation of PRAETORIAN and the interoperability between different CIs. Other participants added to the discussion that the implementation of a holistic system such as PRAETORIAN could also foster the establishment of standardised conditions. On the one hand, PRAETORIAN will likely need to adhere to differing (national or CI-specific) regulations regarding, e.g., data handling, drone neutralisation, or public information distribution. On the other hand, it is also imaginable that some regulations will be adapted in the future in order to enable a holistic, inter-CI security management, which will ideally reach cross-border. Additionally, decision-making responsibilities should be determined, e.g., whether the involvement of higher authorities is needed for risk-management decisions or compliance in information distribution about ongoing situations to the public.

It would therefore be of interest for future research to further evaluate the concept of overarching security management of linked CIs. This should again be accompanied by validations. Based on the conducted validation exercises, some recommendations can be derived.

When evaluating a new system, a challenge lies in the selection of the right end-users for a workplace that does not yet exist. As an example, some of the participants were not ideally suited for their assigned participant roles, e.g., due to limited IT knowledge when evaluating cyber security aspects or were specialists in their fields but unaware of to be considered costs or financial aspects related to system introduction or implementation. In future validations, more and more diverse end-users need to be included to get a more holistic picture. Generally, a bigger sample size would be advisable in order to achieve more representative results.

Furthermore, the applied validation approach was focused on subjective feedback based on the presented scenarios and introduced PRAETORIAN tools, with limited hands-on experiences. Based on this, further developments of the system could be achieved. With increasing maturity, the execution of high-fidelity human-in-the-loop simulations with the fully developed system is recommended. This ensures a higher external validity and also allows for collection of objective performance metrics like response times, from both systems and involved organisations’ personal. Establishing a more realistic environment would enable participants to provide more meaningful assessments of the examined acceptance criteria. Performance data would be especially valuable when evaluating, e.g., in how far PRAETORIAN enables a faster detection of threats (AC A2) or a faster coordinated response (AC B1), but also for other ACs.

Finally, it is noteworthy that the participants proposed several interesting ideas for additional features to be included in the system. This included, e.g., the integration of a function that monitors the current risk level for certain attacks. While a discussion of all proposed ideas is outside the scope of this work, this shows that there are potential features worth exploring in future research.

## Data Availability

The availability of the raw data that was evaluated for this study is available upon request to the corresponding author.
